# Help-Seeking Behaviors and Related Factors in Chinese Patients With Major Depressive Disorder: A Community-Based Cross-Sectional Study

**DOI:** 10.3389/fpsyt.2022.934428

**Published:** 2022-07-06

**Authors:** Xiaojuan Cui, Minghui Li, Peijun Li, Jinhao Li, Xiaofei Hou, Guoli Yan, Peiyao Li, Xuyang Su, Danni Qin, Yijiao Zhang, Yan Gu, Huifang Yin, Guangming Xu

**Affiliations:** ^1^Tianjin Anding Hospital, Mental Health Center of Tianjin Medical University, Tianjin, China; ^2^Institute of Applied Psychology, Tianjin University, Tianjin, China; ^3^Tianjin Third Central Hospital, Tianjin, China

**Keywords:** help-seeking, major depressive disorder, China, non-healthcare sources, healthcare settings, community

## Abstract

**Background:**

Although evidence-based and effective treatments are available for people with major depressive disorder (MDD), a substantial number do not seek or receive help. Therefore, this study aimed to (1) investigate the total help-seeking rate and first-time help-seeking choices; (2) explore the perceived helpfulness of 23 potential sources; and (3) evaluate the factors related to help-seeking behaviors among patients with MDD.

**Materials and Methods:**

Data came from the Tianjin Mental Health Survey (TJMHS), which included a representative sample of adult community residents (*n* = 11,748) in the Chinese municipality of Tianjin. Of these, 439 individuals were diagnosed with lifetime MDD according to the Diagnostic and Statistical Manual-fourth edition (DSM-IV) and administered a help-seeking questionnaire.

**Results:**

In a survey, 28.2% of patients with MDD living community reported that they had ever sought any help during their entire lifetime before the interview, with 8.2% seeking help in mental healthcare settings, 8.0% only in other healthcare settings, and 12.0% only in non-healthcare sources (e.g., family, friends, and spiritual advisor). Among help-seekers, the first help mainly was sought in non-healthcare sources (61.3%), followed by healthcare settings (25.8%) and mental healthcare settings (12.9%). The majority of MDD individuals thought the non-healthcare sources were not helpful and mental healthcare settings were helpful or possibly helpful to solve mental problems. Female, having 10–12 or higher education years, comorbid anxiety disorders were associated with increased help-seeking.

**Conclusion:**

A small percentage of individuals with MDD living in community of Tianjin sought help. They preferred non-healthcare sources to healthcare settings. Demographic and clinical features were associated with help-seeking behaviors.

## Introduction

Major depressive disorder (MDD) is fast becoming one of the most common mental disorders, affecting more than 300 million people worldwide, and has been a leading cause of the burden of disease globally, resulting in serious impairment of functioning and poor quality of life (1, 2). Patients with a hospital encounter for MDD are relatively young, commonly have suicidal ideation/behavior, utilize substantial hospital resources, and have a high risk for a hospital re-encounter in the 30 days post-discharge (3). Compared to outpatients or inpatients, patients with MDD in community are relatively mild (4), and fewer patients in community seek help from healthcare settings (5) and psychiatric medications (6). There is a larger treatment gap in patients with MDD in community. However, the high burden of MDD is due in part to the fact that many people do not receive effective treatment. A recent review, which studied the MDD treatment coverage and gaps in 84 countries from 2000 to 2019 (7), indicated lower treatment rates for health service and mental health services in low- and lower-middle-income countries than high-income countries (for health service: 20 vs. 50%; for mental health service: 8 vs. 33%). It also showed that the treatment rates for the utilization of any service and for any general health service use were 11.6 and 2.3%, respectively, in China. Furthermore, the findings from the China Mental Health Survey (CMHS) suggested a high 12-month prevalence of depressive disorder (3.6%) and low treatment rate (only 9.5%) for patients with 12-month depressive disorders (6, 8, 9). Therefore, it is important to understand the barriers that prevent people with MDD from seeking help, so that targeted interventions can be implemented to improve the wellbeing of those affected, especially in China.

To improve the healthcare of mental disorders, China has undergone a healthcare reform by integrating mental health services into the general healthcare system (10). However, more studies on patients’ perspectives on help-seeking or treatment should be conducted to help identify needs that are not fully met by treatment (11, 12). A patient’s perceived helpfulness of treatment is an important issue, evidenced by the results of the World Mental Health Surveys (WMHS), which showed that 68.2% patients with MDD considered the treatment helpful (13). Moreover, in fact, most residents were more likely to seek help from some non-healthcare sources (such as intimate partner, parents, friends) for depression (14). In China, a previous study provided 23 potential sources for patients with mental disorder to receive help (15) and also showed that Chinese patients with mental disorders were likely to seek different kinds of help-seeking sources and their first-choice helps were sought in non-healthcare sources. In contrast, a study in primary care settings showed that patients with positive Patient Health Questionnaire-9 scores were less willing to take medications or consult a non-medical practitioner, but more likely to consult a psychotherapist (16). Furthermore, previous studies have identified some factors that influence help-seeking behaviors, such as some demographic factors, illness phases, health policies, and mental health resources (17–19), but most of them mainly focused on demographic factors ignoring clinical issues such as severity and comorbidity. To our knowledge, few research has been conducted to evaluate the help-seeking patterns in Chinese patients with MDD in population surveys. Even in the recent review, only three studied in MDD were included and they only reported the total treatment rates in a variety of healthcare and non-healthcare sources (7).

To explore more information about the help-seeking patterns of patients with MDD in a population survey, we conducted this study based on the data from the Tianjin Mental Health Survey (TJMHS). The objectives were to explore (1) the help-seeking rate and first-time help-seeking proportion from 23 potential sources; (2) the perceived helpfulness of 23 potential sources in a representative sample of individuals with a lifetime diagnosis of *Diagnostic and Statistical Manual-fourth edition* (DSM-IV) MDD; and (3) the clinical (such as severity, comorbidity) and demographic factors associated with help-seeking among patients with MDD.

## Materials and Methods

### Sample and Procedures

Data came from the TJMHS conducted from July 2011 to March 2012, including a large representative adult community population in Tianjin Municipality (*n* = 11,748). A two-phase design and a multistage cluster random sampling method were used in this survey. In the first screening phase, the expanded version of the 12-item General Health Questionnaire (GHQ-12) was used to determine the psychopathological risk of 11,748 subjects. The second phase of diagnosis involves performing SCID to determine whether there is a DSM-IV diagnosis. According to the screening results, interviewees were classified as having one of the three risks of a mental disorder (low-risk, medium-risk, and high-risk). All high-risk subjects and random samples from medium-risk subjects (45.7%) and low-risk subjects (11.5%) were selected for the diagnostic phase. Of the 4,563 selected respondents, 4,438 completed the interview with the Structured Clinical Interview for the DSM-IV axis I disorders (SCID), and a help-seeking questionnaire (see below). Finally, among the 4,438 subjects, 439 individuals met the criteria for lifetime MDD. More detailed information was illustrated in our previous study (20, 21).

A total of 44 psychiatrists were recruited as interviewers to perform the two phases. They all received 21 days’ rigorous training. All subjects were interviewed face-to-face at participants’ homes. An interviewer read the scales to participants because of the difficulty for some individuals to complete these scales on their own. The psychiatrists were allowed to explain the meaning of the standard questions in the scales when participants did not understand the meaning of questions. The Medical Ethics Committee of Tianjin Mental Health Center approved the research protocol. Prior to participation, all interviewees signed declaration of consent.

### Measures

Demographic and clinical information about the participants with MDD were derived from TJMHS. Demographic information included gender (male or female), age (18–39, 40–54, 55+ years), resident region (urban or rural area), years of education (0–6, 7–9, 10–12, 13+), marital status (never married/divorced/lost spouse or married), employment status (housewife, unemployed/lost job/retired, or employed), income group (above median, do not know, or below median), and living status (live alone or live with other people). In addition, clinical information comprised the severity of MDD (mild, moderate, or severe), age of onset, and comorbidity with other mental disorders.

### The 12-Item General Health Questionnaire

The Chinese version of the GHQ-12 (22) consisted of 12 items. It was used to assess overall mental distress during the past month. The answers to each question are as follows: 0 = “better than usual,” 0 = “as usual,” 1 = “less than usual,” and 1 = “much less than usual,” and the score is between 0 and 12. A GHQ-12 score of at least 4 indicated having mental health problems. A previous study suggested that the scale should have sufficient test–retest reliability (0.72) and internal consistency (Cronbach’s α = 0.75) (22). In this study, the scale has good internal consistency (Cronbach’s α = 0.90).

### Structured Clinical Interview for the Diagnostic and Statistical Manual

Based on the Chinese version of the DSM-IV SCID, the diagnosis of MDD and multiple comorbid diagnoses were completed (23). If there were multiple diagnoses, the examiner should rank them according to their clinical significance. In this study, besides MDD, comorbid diagnoses included (1) anxiety disorders, including panic disorder, agoraphobia without panic, social phobia, specific phobia, obsessive-compulsive disorder, post-traumatic stress disorder, generalized anxiety disorder, and not otherwise specified anxiety disorders; (2) substance-use disorders, including alcohol-use and sedative/hypnotic drug-use disorders; (3) organic mental disorders, including intellectual disability, dementia, and mental disorders due to a general medical condition, or due to substance use. In this study, “lifetime” means meeting diagnostic criteria at any time in the individual’s lifetime and “current” means meeting diagnostic criteria at any time in the previous month. The Chinese SCID has proven to be valid and reliable (24). All participants were interviewed by professionally trained psychiatrists.

### Help-Seeking Questionnaire

Our study used a detailed help-seeking questionnaire to assess subjects’ help-seeking behaviors for mental problems. The questionnaire was developed by Michael Phillips (23). About 23 sources of help for mental health issues were listed in the questionnaire. For each help-seeking source, participants were asked if they had used it (answering “yes”/“no”) and their perceived helpfulness on these sources. If the individual reported that they never sought help from any, they were only asked whether they rated it helpful to deal with mental problems for each source. In this study, the 23 help-seeking sources were divided into healthcare settings and non-healthcare sources. Healthcare settings included non-mental healthcare settings and mental healthcare settings. The former contained a private doctor of Traditional Chinese Medicine (TCM), a private doctor of Western medicine, inpatient treatment in a TCM hospital, an outpatient clinic in a TCM hospital, a neurology clinic in a general hospital, an internal medicine clinic in a general hospital, inpatient treatment in a general hospital, a community pharmacy, and a community health center. The latter consisted of a psychiatric clinic in a general hospital, a specialized clinic in a psychiatric hospital, a regular clinic in a psychiatric hospital, inpatient treatment in a psychiatric hospital, and a community psychotherapy institute. The non-healthcare sources included relatives, colleagues/friends/neighbors, a Qigong practitioner, a witch doctor, a temple, writing letters to get counseling, a newspaper article or magazine, an Internet support group, and a hotline. When interviewees said they received two or more sources of help, they were asked what kind of source they sought first.

### Global Assessment of Functioning

The same psychiatrist who administered the SCID used GAF to assess the level of dysfunction due to mental illness in the previous month (25). The score of GAF scale is 1–100, with higher scores representing better functioning. GAF score is used to evaluate the severity of disability weight [disability weight = (100 − GAF score)/100]. We categorized severity into mild disabled (with a disability weight of < 0.40) and moderately to severely disabled (with a disability weight of ≥ 0.4).

### Statistical Analyses

Analyses were performed using SPSS 25.0, and a significance level of *p* < 0.05 was used in all analyses. The participants were initially divided into groups of those seeking help and those who were not. Means and standard deviations or frequencies and percentages were used to describe the demographic and clinical variables. The chi-square test was used to compare the differences between help-seeking group and non-help-seeking group. To identify factors associated with help-seeking behaviors, univariate and multivariate logistic regression analyses were performed. All factors that attained a significance level of *p* < 0.05 in univariate analysis were included in the multivariate analysis. Typical regression assumptions (e.g., linearity and collinearity) were tested, and there were no violations.

## Results

### Characteristics of the Study Sample

There were 439 individuals diagnosed with MDD. The sample included 314 women (71.5%) and 125 men (28.5%). Study participants had a mean age of 54.38 (SD = 13.9) years. Before the interview, 28.9% of the participants reported lifetime history of comorbid other mental disorders (*n* = 127). The results showed that there were statistical differences between the help-seeking group and non-help-seeking group in terms of gender (χ^2^ = 9.773, *p* = 0.002), age (χ^2^ = 8.454, *p* = 0.015), education years (χ^2^ = 14.989, *p* = 0.002), and whether they had comorbid anxiety disorders (χ^2^ = 20.270, *p* < 0.001). Table 1 provides details of the characteristics of the research sample.

**TABLE 1:** Characteristics for participants with MDD who did (*N* = 124) and did not (*N* = 315) seek help.

Characteristics		Total n (%)	Help-seeking n (%)	Non-help-seeking n (%)	χ^2^	*p*
**Demographic characteristics**						
Gender	Male	125 (28.5)	22 (17.7)	103 (32.7)	9.773	0.002**
	Female	314 (71.5)	102 (82.3)	212 (67.3)		
Resident region	Rural	138 (31.4)	34 (27.4)	104 (33.0)	1.293	0.256
	Urban	301 (68.6)	90 (72.6)	211 (67.0)		
Age range	55+	231 (52.6)	54 (43.5)	177 (56.2)	8.454	0.015*
	40–54	137 (31.2)	41 (33.1)	96 (30.5)		
	18–39	71 (16.2)	29 (23.4)	42 (13.3)		
Years of education	0–6	174 (39.6)	39 (31.5)	135 (42.9)	14.989	0.002**
	7–9	138 (31.4)	34 (27.4)	104 (33.0)		
	10–12	79 (18.0)	28 (22.6)	51 (16.2)		
	13+	48 (10.9)	23 (18.5)	25 (7.9)		
Marital status	Married	295 (67.2)	83 (66.9)	212 (67.3)	0.005	0.941
	Never married/divorce/lose spouse	144 (32.8)	41 (33.1)	103 (32.7)		
Employment status	Unemployed/lost job/retired	205 (46.7)	49 (39.5)	156 (49.5)	3.704	0.157
	Employed	182 (41.5)	57 (46.0)	124 (39.4)		
	Housewife	53 (12.1)	18 (14.5)	35 (11.1)		
Income group	Below median or do not know	272 (61.9)	71 (57.3)	201 (63.8)	1.620	0.203
	Above median	167 (38.1)	53 (42.7)	114 (36.2)		
Living status	Alone	86 (19.6)	19 (15.3)	67 (21.3)	1.998	0.158
	Not alone	353 (80.4)	105 (84.7)	248 (78.7)		
**Mental health characteristics**						
GHQ-12 score	<4	204 (46.5)	55 (44.4)	149 (47.3)	1.755	0.185
	≥4	235 (53.5)	69 (55.6)	166 (52.7)		
GAF disability	Moderate to severe	156 (35.5)	43 (34.7)	113 (35.9)	0.089	0.766
	Mild	283 (64.5)	81 (65.3)	202 (64.1)		
Comorbid anxiety disorders	No	393 (89.5)	98 (79)	295 (93.7)	20.270	< 0.001**
	Yes	46 (10.5)	26 (21)	20 (6.3)		
Comorbid substance use disorders	No	400 (91.1)	113 (91.1)	287 (91.1)	<0.001	0.995
	Yes	39 (8.9)	11 (8.9)	28 (8.9)		
Comorbid organic mental disorders	No	423 (96.4)	118 (95.2)	305 (91.8)	0.702	0.402
	Yes	16 (3.6)	6 (4.8)	10 (3.2)		
Age of onset^#^	55+	135 (31.3)	31 (25.0)	104 (33.0)	3.444	0.179
	40–54	147 (34.1)	42 (33.9)	105 (33.3)		
	18–39	149 (34.6)	49 (39.5)	100 (31.7)		

^#^Percentages do not sum to 100% due to missing data (total sample is 431, and there is 8 missing data). *p < 0.05, **p < 0.01.

### Help-Seeking Patterns of Major Depressive Disorder Respondents

The rates of help-seeking of patients with MDD are shown in Table 2. In the total sample (*N* = 439), 28.2% of patients with MDD reported that they had used any source before the interview.

**TABLE 2:** The help-seeking rates (*N* = 439) and the proportion of first help (*N* = 124) for 23 sources in MDD individuals.

	Help seeking sources	Help-seeking rates (*N* = 439)		Proportion of first help (*N* = 124)	
			
		*n*	%	*n*	%
1	Relatives	53	12.1	43	34.7
2	Colleagues/friends/neighbors	54	12.3	27	21.8
3	A private doctor of western medicine	6	1.3	2	1.6
4	A private doctor of Traditional Chinese Medicine (TCM)	4	0.9	2	1.6
5	A witch doctor	8	1.8	2	1.6
6	A Qigong practitioner	2	0.4	0	0
7	An internal medicine clinic in a general hospital	16	3.6	7	5.7
8	A neurology clinic in general hospital	6	1.4	3	2.4
9	A psychiatric clinic in a general hospital	14	3.2	5	4.0
10	Inpatient treatment in a general hospital	2	0.5	1	0.8
11	An outpatient clinic in a TCM hospital	18	4.1	11	8.9
12	Inpatient treatment in a TCM hospital	0	0	0	0
13	A regular clinic in a psychiatric hospital	19	4.3	9	7.3
14	A specialized clinic in a psychiatric hospital	2	0.5	0	0
15	Inpatient treatment in a psychiatric hospital	4	0.9	1	0.8
16	A community psychotherapy institute	2	1.6	1	0.8
17	A community health center	5	4.0	4	3.2
18	A community pharmacy	2	0.5	2	1.6
19	A temple	0	0	0	0
21	Writing letters to get counseling	0	0	0	0
21	A newspaper article or magazine	2	0.5	2	1.6
22	An internet support group	0	0	0	0
23	A hotline	0	0	0	0
24	Other	2	0.5	2	1.6
Any form of help*^a^*		124	28.2	124	100
Any healthcare settings*^b^*		71	16.2	48	38.7
Any mental healthcare settings*^c^*		36	8.2	16	12.9
Only non-mental healthcare settings*^d^*		35	8.0	32	25.8
Only non-healthcare sources*^e^*		53	12.0	76	61.3

*^a^Includes all forms of help listed in the table.*

*^b^Includes mental healthcare settings and non-mental healthcare settings.*

*^c^Mental healthcare settings include a psychiatric clinic in a general hospital, a regular clinic in a psychiatric hospital, a specialized clinic in a psychiatric hospital, inpatient treatment in a psychiatric hospital, and a community psychotherapy institute.*

*^d^Non-mental healthcare settings include a private doctor of western medicine, a private doctor of Traditional Chinese Medicine (TCM), an internal medicine clinic in a general hospital, a neurology clinic in general hospital, inpatient treatment in a general hospital, an outpatient clinic in a TCM hospital, inpatient treatment in a TCM hospital, a community health center, and a community pharmacy.*

*^e^Non-healthcare sources include relatives and colleagues/friends/neighbors, a witch doctor, a Qigong practitioner, a temple, writing letters to get counseling, a newspaper article or magazine, an Internet support group, and a hotline.*

Also, 8.2% of patients with MDD use mental healthcare settings, and 8.0% of patients with MDD use only non-mental healthcare settings. About 12.0% of patients with MDD only sought help from non-healthcare sources. The most common source was colleagues/acquaintances/neighbors (12.3%), followed by relatives (12.1%). A regular clinic in a psychiatric hospital (4.3%), an outpatient clinic in a TCM hospital (4.1%), a community health center (4.0%), an internal medicine clinic in a general hospital (3.6%), and a psychiatric clinic in a general hospital (3.2%) were the five most commonly used healthcare settings.

Most of the patients with MDD with help-seeking behaviors first sought help from relatives and colleagues/friends/neighbors (56.5%) and non-healthcare sources (61.3%). Approximately 25.8% sought help from non-mental healthcare settings first and 12.9% sought mental healthcare settings first. In terms of healthcare settings, the most common first choice was an outpatient clinic in a TCM hospital (8.9%), followed by a regular clinic in a psychiatric hospital (7.3%), an internal medicine clinic in a general hospital (5.7%), a psychiatric clinic in a general hospital (4.0%), and a community health center (3.2%).

### Attitudes Toward the Helpfulness of Different Help-Seeking Sources

Table 3 provides a detailed description of respondents’ attitudes toward different help-seeking sources. Among patients with MDD who had never sought help, 51.2–97% of patients with MDD rated the non-healthcare pathways as unhelpful in addressing mental health problems, the highest percentage was 97% for a Qigong practitioner, followed by 96.9% for a witch doctor, 91.2% for a temple, 81.9% for writing letters to get counseling, 79.1% for an Internet support group, 78.2% for a hotline, 78.1% for a newspaper article, 52.2% for colleagues/friends/neighbors, and 51.2% for relatives; about 64.1–86.3% of patients with MDD rated the non-mental healthcare settings unhelpful, the highest percentage were 86.3% for a community pharmacy, followed by 83.1% for a private doctor of Western medicine, 82.7% for a community health center, 80.2% for a private doctor of TMC, 71.2% for an internal medicine clinic in a general hospital, 68.5% for inpatient treatment in a TCM hospital, 67.9% for inpatient treatment in a general hospital, 67.4% for an outpatient clinic in a TCM hospital, and 64.1% for a neurology clinic in general hospital. Also, 41.6–55.5% of patients with MDD rated mental healthcare settings unhelpful, the highest percentage was 55.5% for a community psychotherapy institute, followed by 46.2% for inpatient treatment in a psychiatric hospital, 44.7% for a regular clinic in a psychiatric hospital, and 41.6 for a specialized clinic in a psychiatric hospital.

**TABLE 3:** Attitudes toward helpfulness of different help-seeking sources among people with MDD who never used and ever used each source.

Help seeking sources		Never used				Used			
			
		*N*	Unhelpful n (%)	Possible helpful n (%)	Definitely helpful n (%)	*N*	Unhelpful n (%)	Possible helpful n (%)	Definitely helpful n (%)
1	Relatives*^a^*	381	195 (51.2)	116(30.4)	70(18.4)	51	4 (7.8)	17 (33.3)	30 (58.8)
2	Colleagues/friends/neighbors*^a^*	379	198 (52.2)	116(30.6)	65(17.2)	53	4 (7.5)	19 (35.8)	30 (56.6)
3	A private doctor of western medicine*^b^*	426	354 (83.1)	53(12.4)	19(4.5)	6	2 (33.3)	1 (16.7)	3 (50.0)
4	A private doctor of Traditional Chinese Medicine (TCM)*^b^*	429	344 (80.2)	63(14.7)	22(5.1)	3	2 (66.7)	0	1 (33.3)
5	A witch doctor*^a^*	424	411 (96.9)	11(2.6)	2(0.5)	8	4 (50.0)	3 (37.5)	1 (12.5)
6	A Qigong practitioner*^a^*	430	417 (97.0)	13(3.0)	0	2	2 (100.0)	0	0
7	An internal medicine clinic in a general hospital*^b^*	416	296 (71.2)	90(21.6)	30(7.2)	16	5 (31.3)	5 (31.3)	6 (37.5)
8	A neurology clinic in general hospital*^b^*	426	273 (64.1)	112(26.3)	41(9.6)	6	1 (16.7)	2 (33.3)	3 (50.0)
9	A psychiatric clinic in a general hospital*^c^*	418	188 (45.0)	145(34.7)	85(20.3)	14	2 (14.3)	0	12 (85.7)
10	Inpatient treatment in a general hospital*^b^*	430	292 (67.9)	90(20.9)	48(11.2)	2	0	0	2 (100.0)
11	An outpatient clinic in a TCM hospital*^b^*	414	279 (67.4)	102(24.6)	33(8.0)	18	4 (22.2)	4 (22.2)	10 (55.6)
12	Inpatient treatment in a TCM hospital*^b^*	432	296 (68.5)	98(22.7)	38(8.8)	0	0	0	0
13	A regular clinic in a psychiatric hospital*^c^*	414	185 (44.7)	133(32.1)	96(23.2)	18	5 (27.8)	0	13 (72.2)
14	A specialized clinic in a psychiatric hospital*^c^*	430	179 (41.6)	145(33.7)	106(24.7)	2	1 (50.0)	0	1 (50.0)
15	Inpatient treatment in a psychiatric hospital*^c^*	429	198 (46.2)	126(29.4)	105(24.5)	3	0	0	3 (100.0)
16	A community psychotherapy institute*^c^	429	238 (55.5)	116(27.0)	75(17.5)	2	1 (50.0)	0	1 (50.0)
17	A community health center*^b^*	427	353 (82.7)	57(13.3)	17(4.0)	5	0	1 (20.0)	4 (80.0)
18	A community pharmacy*^b^*	430	371 (86.3)	45(10.5)	14(3.3)	2	0	0	2 (100.0)
19	A temple*^a^*	432	394 (91.2)	34(7.9)	4(0.9)	0	0	0	0
20	Writing letters to get counseling*^a^	431	353 (81.9)	64(14.8)	14(3.2)	0	0	0	0
21	A newspaper article or magazine*^a^*	430	336 (78.1)	73(17.0)	21(4.9)	2	0	1 (50.0)	1 (50.0)
22	An internet support group*^a^	431	341 (79.1)	74(17.2)	16(3.7)	0	0	0	0
23	A hotline*^a^	431	337 (78.2)	85(19.7)	9(2.1)	0	0	0	0

*Indicates that one individual did not report the attitude toward it. ^a^Indicates non-healthcare sources. ^b^Indicates non-mental healthcare settings. ^c^Indicates mental healthcare settings.

Among patients with MDD who had ever sought any help, 0–100% of patients with MDD thought the non-healthcare pathways unhelpful in addressing mental health problems, with the highest percentage 100% for a Qigong practitioner. Also, 0–66.7% of patients with MDD rated the non-mental healthcare settings unhelpful, with the highest percentage of 66.7% for a private doctor of TCM. About 14.3–50.0% of patients with MDD assessed mental healthcare settings unhelpful with the highest percentage 50.0% for a specialized clinic in a psychiatric hospital.

### Factors Related to Help-Seeking Behaviors

Univariate and multivariate logistic regression analyses were conducted to examine the factors related to help-seeking. The results are shown in Table 4. Several variables were associated with greater rate of help-seeking: women (OR = 2.36, 95% CI = 1.37–4.07), 10–12 or more than 13 years of education (compared with 0–6 years of education) (10–12 years of education, OR = 2.06, 95% CI = 1.08–3.91; 13+ years of education, OR = 2.92, 95% CI = 1.35–6.33), comorbid anxiety disorders (OR = 3.51, 95% CI = 1.81–6.79). Generally, women, having 10–12 or 13+ years of education and reporting comorbid anxiety disorders, were associated with higher rate of help-seeking.

**TABLE 4:** Association between demographic and clinical characteristics and help-seeking (yes/no) in individuals with MDD.

Characteristics		Univariable, OR (95% CI)	*p*	Multivariable, OR (95% CI)	*p*
Sex	Male	1		1	
	Female	2.25 (1.34–3.78)	**0.002**	**2.40 (1.36–4.22)**	**0.003**
Resident area	Rural	1		-	-
	Urban	1.31 (0.82–2.07)	0.256	-	-
Age range	55+	1		1	
	40–54	1.40 (0.87–2.25)	0.166	0.93 (0.50–1.72)	0.805
	18–39	**2.26 (1.29–3.97)**	**0.004**	1.25 (0.57–2.73)	0.583
Years of education	0–6	1		1	
	7–9	1.13 (0.67–1.92)	0.645	1.28 (0.69–2.38)	0.434
	10–12	**1.90 (1.06–3.40)**	**0.031**	**2.08 (1.04–4.15)**	**0.037**
	13+	**3.19 (1.63–6.22)**	**0.001**	**2.72 (1.19–6.20)**	**0.017**
Marital status	Married	1		-	-
	Never married/divorce/lose spouse	1.02 (0.65–1.58)	0.941	-	-
Employment status	Unemployed/lost job/retired	1		-	-
	Employed	1.46 (0.93–2.29)	0.096	-	-
	Housewife	1.64 (0.85–3.15)	0.139	-	-
Income group	Below median or do not know	1		-	-
	Above median	1.32 (0.86–2.01)	0.204	-	-
Living status	Alone	1		-	-
	Not alone	1.49 (0.85–2.61)	0.159	-	-
GHQ-12 score	<4	1		-	-
	≥4	1.33 (0.87–2.01)	0.186	-	-
GAF disability	Moderate to severe	1		-	-
	Mild	1.05 (0.68–1.63)	0.814	-	-
Comorbid anxiety disorders	No	1			
	Yes	**3.91 (2.09–7.32)**	**<0.001**	**3.76 (1.91–7.38)**	**<0.001**
Comorbid substance use disorders	No	1		-	-
	Yes	1.00 (0.48–2.07)	0.995	-	-
Comorbid psychotic disorders	No	1		-	-
	Yes	3.88 (0.64–23.51)	0.140	-	-
Age at first onset	55+	1		-	-
	40–54	1.34 (0.78–2.30)	0.284	-	-
	18–39	1.64 (0.97–2.78)	0.064	-	-

*Confidence intervals and p-values in bold type as statistically significant at the p < 0.05 level.*

## Discussion

To the best of our knowledge, this is the first study to describe the perceived helpfulness of about 23 sources, the help-seeking rates, and its correlation in the Chinese community patients with MDD. This study found that the total rate of help-seeking was 28.2%. Non-healthcare sources were the main sources and the first choice for help-seekers with major depression. Sex, years of education, and comorbid anxiety disorders were rated factors for help-seeking behaviors.

The help-seeking rate in this study is lower compared with the results of previous studies. A study in China found that 61% of individuals with major depressive episodes sought any help, which is higher than our results (5). In the Netherlands, a survey showed that 65% of patients with MDD had sought help in the past 6 months (26). A survey in Estonia suggested that the rate of 12-month help-seeking was 34.1% (27). Although the methodological methods such as diagnostic time frame, different assessment questionnaires, and sample characteristics may be the cause of this inconsistency, it also indicated the need to cover the treatment gap for patients with MDD. For the healthcare settings, the rate (16.2%) in this study is similar to previous survey results in Tianshui City, Gansu Province (16.13%) (28), and is higher than the survey results from Hebei Province (13.0%) (29). However, this proportion is much lower than in Western countries (30.6% in Turkey and 34% in Finland) (30, 31). The difference of rates of help-seeking between mainland China and Western countries may be caused by various factors such as relatively low mental health literacy and insufficient mental health resources and the psychiatric workforce in China compared to Western countries (32, 33). In a nutshell, only a small percentage of people turn to healthcare settings for help.

Earlier studies have shown that people’s attitudes about seeking help, as well as preferences and beliefs about treatment, have a strong influence on their help-seeking behaviors (34, 35). In this study, among those who had not sought help, the majority did not think the help-seeking sources as useful in managing mental problems. A study from the WMHS in 16 countries showed that 68.2% of adults with MDD who had received treatment reported that they perceived it to be effective (13). This calls for improving public mental health literacy and enriching mental healthcare resources for enhancing the accessibility of depression care.

Most patients with MDD choose non-healthcare sources as the main sources of help. Relatives and colleagues/friends/neighbors were the first options for help-seeking and the most frequently mentioned sources, which was in line with former studies. A study from TJMHS showed that relatives or friends was the first choice among people with mental disorders (15). Another study showed that residents with depressive symptoms preferred to seek help from non-healthcare sources (e.g., friends, parents) (36). One possible explanation is that they are easier to access. In addition, they are usually reliable sources of support, especially emotional and informational support, and may help patients with MDD to seek help from healthcare settings (14). Relying on these helps to reduce stigmatization is another explanation (37). Depression itself can cause communication difficulties. The perceived trust and non-judgmental attitude can promote communication and make it easier for people with depression to seek help.

However, for the folk sources, like Qigong and a witch doctor, this study showed that only 2.2% sought help through these sources, and 1.6% sought them first. Intriguingly, these rates were much lower than preceding studies in the 1990s, which showed that 70.5% of psychiatric patients in rural communities and 73.9% of psychiatric outpatients from rural areas received consultation with witch doctors (38). To seek help from a witch doctor was based on the belief that mental illness was caused by evil spirits invading one’s body or punishing someone or their ancestors for doing wrong (39). However, this study showed that folk sources might be losing their role in dealing with mental problems.

The finding that TCM plays an important role is consistent with the prior study. A study unanimously found that some Chinese were more willing to seek help from TCM (40). The possible reason may be that some people with depression will experience non-specific physical symptoms (e.g., weight loss and body pain) (41). Hence, the holistic (the whole body) perspective in TCM may prompt patients to seek help from these settings. In this study, only a very small number of patients with MDD went to community healthcare settings (a community health center, a community psychotherapy institute, a community pharmacy). Finally, before seeking help from a psychiatrist, they can seek help from various sources. Therefore, more projects need to be carried out to improve the detection of mental health problems in TCM and community healthcare settings.

Most patients with MDD first visited non-mental healthcare settings (25.8%) rather than mental healthcare settings (12.9%). The finding is consistent with another study (15). Three factors accounted for these circumstances. First, a representative survey of mental health literacy in China showed low mental health literacy (33). People with low mental health literacy were more likely to reduce mental healthcare use and delay medical treatment options (42). Second, due to the stigma of mental illness and the fear of discrimination, they prefer general hospitals to mental health settings (43). Third, this result may stem from the limited availability of psychiatrists and psychiatric hospitals. Mental health services were mainly focused on large psychiatric hospitals in major cities in central and eastern China (44). Thus, it is essential to strengthen the application of integrated non-mental healthcare settings in MDD treatment (10).

In this study, women sought help of any kind more often than men. The same association between gender and help-seeking was also shown in another study (45). Women have lower depression stigma than men and might be more willing to share and identify psychological distress (46). In addition, traditional ideas of masculinity were often seen as a cause of this phenomenon. For men, receiving support or seeking help was associated with the risk of being marginalized or as well as being seen as “unmanly” by others (47). Furthermore, the sample in this study consisted of more women than men, which might cause bias in the results. However, considering the current and previous findings, special attention should be paid for educating men to seek help when they were found to have MDD.

People with higher education were more likely to seek help in this research, which is in line with the previous study (48). This can be explained by stigmatization: people with higher education were less stigmatized (49). Thus, they were often willing to admit having mental health problems and seek help. Apart from stigma, this result also could be explained by mental health literacy. Many people did not have a proper understanding of MDD, which prevented them from seeking help in this regard. A study showed that individuals with higher education reported greater depression knowledge and mental health literacy, and they are more likely to perceive the need for help-seeking (50).

Patients reporting comorbidity between MDD and anxiety disorders were more likely to seek help from any source. This is similar to previous studies. A study reported that a higher likelihood of service use was found among those who had one mental disorder comorbid with depression (51). A study in Finland showed that comorbid generalized anxiety disorder was positively related to help-seeking behaviors in patients with MDD (31). Help-seeking with depression and other mental health issues is believed to go through several phases, including subjective experience of symptoms, weighing their importance and possible consequences, assessing whether the symptoms require intervention, and evaluating the costs and benefits of various options (52). Therefore, patients reporting comorbid mental disorders need to intervene at the beginning of the process.

There are certain restrictions that need to be considered. First, help-seeking behaviors were evaluated based on the interviewee’s self-report and might be subject to recall bias. Second, given the cross-sectional nature of the data, it is impossible to examine the causal relationship between help-seeking behaviors and other variables. Third, the current research findings are especially applicable to the Tianjin area, and the research findings should be carefully generalized to other regions/countries. Nonetheless, findings can show patterns of help-seeking and their correlations that would be found in similar regions with rapidly changing socio-economic conditions. Fourth, we cannot compare the difference of the attitudes to each source between the patients who have ever sought help and the patients who never sought help because the small number of individuals who have ever sought each help. Fifth, factors (such as stigma and suicidal acts) that may affect help-seeking behaviors were not included in this study; therefore, future research should include more variables to explore the factors related to help-seeking behaviors.

Despite these limitations, there are several strengths. First, the samples in this study were individuals with MDD diagnosed by SCID rather than persons with depressive symptoms. This made the results more valuable to provide detailed information for the healthcare system for patients with MDD. Second, the help-seeking rates and perceived helpfulness of 23 potential sources evaluated in this study could provide a more comprehensive picture of help-seeking behaviors among patients with MDD. Third, this study considered not only sociodemographic factors but also clinical factors on help-seeking behaviors. This study contributed to more evidence-based examination of the factors related to help-seeking behaviors in a sample of the community.

## Conclusion

This study shows that only a small proportion of people with MDD sought help. For women, more education attainment and comorbid anxiety disorders were positively associated with help-seeking behaviors for patients with MDD. More sources should be considered to be as help providers such as non-healthcare sources, and increasing the perceived helpfulness of healthcare settings in patients with MDD is an important issue to promote community residents to actively seek healthcare settings.

## Data Availability Statement

The original contributions presented in this study are included in the article/supplementary material, further inquiries can be directed to the corresponding authors.

## Ethics Statement

The studies involving human participants were reviewed and approved by the Medical Ethics Committee of Tianjin Mental Health Center. The patients/participants provided their written informed consent to participate in this study.

## Author Contributions

GX, HY, XC, and ML designed the study and wrote the manuscript. XC, ML, and PJL managed the literature searches and analyses. PYL, XS, DQ, and YZ provided data curation. JL, XH, YG, and GY undertook the statistical analysis. All authors critically reviewed the draft and helped to revise the manuscript.

## Conflict of Interest

The authors declare that the research was conducted in the absence of any commercial or financial relationships that could be construed as a potential conflict of interest.

## Publisher’s Note

All claims expressed in this article are solely those of the authors and do not necessarily represent those of their affiliated organizations, or those of the publisher, the editors and the reviewers. Any product that may be evaluated in this article, or claim that may be made by its manufacturer, is not guaranteed or endorsed by the publisher.
